# Factors Related to Women’s Psychological Distress during the COVID-19 Pandemic: Evidence from a Two-Wave Longitudinal Study

**DOI:** 10.3390/ijerph182111656

**Published:** 2021-11-06

**Authors:** Maria Di Blasi, Gaia Albano, Giulia Bassi, Elisa Mancinelli, Cecilia Giordano, Claudia Mazzeschi, Chiara Pazzagli, Silvia Salcuni, Gianluca Lo Coco, Omar Carlo Gioacchino Gelo, Gloria Lagetto, Maria Francesca Freda, Giovanna Esposito, Barbara Caci, Aluette Merenda, Laura Salerno

**Affiliations:** 1 Department of Psychology, Educational Science and Human Movement, University of Palermo, 90128 Palermo, Italy; cecilia.giordano@unipa.it (C.G.); gianluca.lococo@unipa.it (G.L.C.); barbara.caci@unipa.it (B.C.); aluette.merenda@unipa.it (A.M.); laura.salerno@unipa.it (L.S.); 2 Department of Developmental and Social Psychology, University of Padova, 35132 Padova, Italy; giulia.bassi@phd.unipd.it (G.B.); elisa.mancinelli@phd.unipd.it (E.M.); silvia.salcuni@unipd.it (S.S.); 3 Digital Health Lab, Fondazione Bruno Kessler, 38122 Trento, Italy; 4 Department of Philosophy, Social & Human Sciences and Education, University of Perugia, 06123 Perugia, Italy; claudia.mazzeschi@unipg.it (C.M.); chiara.pazzagli@unipg.it (C.P.); 5 Department of History, Society and Human Studies, University of Salento, 73100 Lecce, Italy; omar.gelo@unisalento.it (O.C.G.G.); gloria.lagetto@unisalento.it (G.L.); 6 Faculty of Psychotherapy Science, Sigmund Freud University Vienna, 1020 Vienna, Austria; 7 Department of Humanities, University of Napoli Federico II, 80133 Napoli, Italy; mariafrancesca.freda@unina.it (M.F.F.); giovan.esposito@unina.it (G.E.)

**Keywords:** women, COVID-19, distress, principal component analysis, emotion regulation, social stability status, intolerance of uncertainly

## Abstract

Background. A growing body of research has highlighted the negative effects of the COVID-19 pandemic on women’s mental health. Previous studies showed that women have higher levels of depression, anxiety and PTSD, and worse psychological adjustment than men, which also persisted after the earlier phase of the pandemic. This study aimed to evaluate changes in women’s psychological distress during the pandemic and to evaluate the factors that have a more significant impact in predicting women’s psychological distress. Methods. This two-wave longitudinal study (T1 = Italian first lockdown, and T2 = second phase, when the restrictive measures were eased) involved 893 women (M_age_ = 36.45, SD = 14.48). Participants provided demographic and health data as well as measures of psychological distress, emotion regulation processes, and ability to tolerate uncertainty. Results. No significant changes were found in women’s psychological distress between T1 and T2, i.e., during and after the first lockdown. Lower social stability status and higher maladaptive emotional coping predicted high psychological distress. Conclusions. Results showed that modifiable psychological variables play a central role in predicting distress and indicated that emotion regulation interventions might be helpful in increasing psychological resilience and mitigating the adverse impacts of the COVID-19 pandemic within the female population.

## 1. Introduction

The worldwide spread of COVID-19 and subsequent mitigation measures have caused a significant increase in mental health problems in many countries.

In addition to the primary threat of infection, social distancing measures to combat the spread of the virus have also had negative psychological, social, and economic consequences on the general population [1,2]. Relevant reviews of the consequences of the COVID-19 pandemic on mental health have evidenced increased levels of stress, anxiety and depression, as well as an increase in suicidal ideation across several countries [3,4,5]. Italy is one of the European countries that have been most dramatically affected by COVID-19. From 9 March 2020 until the 4th of May, the Italian government implemented extraordinary measures to limit the viral transmission, which severely restricted movement of individuals across the whole nation and imposed severe social distancing restrictions. Later, during the second phase, although a large number of preventive and protective measures were still adopted, the mass quarantine was lifted, and restrictive measures were eased. Previous studies on the Italian population showed that, as in the rest of the world, there were high levels of psychological distress and negative mental health effects as a result of the current health emergency [6,7,8]. Several risk factors, including job loss and economic hardship, reduced sources of social support, increases in familiar disorders and violence, have been linked to worsening levels of mental health during the COVID-19 pandemic [9,10,11]. Moreover, a growing body of research has also evidenced a more negative mental health impact of the COVID-19 pandemic within specific, vulnerable groups such as women, the young, people with lower educational levels, lower-income, or pre-existing mental health conditions [12,13,14,15]. Previous studies on predictors of mental health during COVID-19 evidenced that women had the highest prevalence of mental health problems during the first period of the pandemic [16,17].

In line with these research findings, this study aimed to examine the effects of the pandemic on women’s mental health, in order to generate mitigating action that might prevent/alleviate the psychological consequences of the pandemic on women.

Significant gendered pandemic effects may have a negative impact on women’s health globally. For example, a greater rate of unemployment among women compared to men during the outbreak has had a detrimental effect on their work and economic opportunities, and increased the pressure to take on caring roles within families as schools and elderly care facilities close [18,19]. Although mortality rates have been twice as high for men as for women [20], the COVID-19 pandemic has affected women’s mental health more than men. Several studies highlighted the fact that the increase in women’s distress is, in part, due to exacerbated domestic duties, including childcare, linked to working from home and school closures [12,21,22,23]. This is particularly relevant in the Italian context, where the COVID-19 pandemic has increased the focus on social and cultural inequalities concerning gender, which place Italian women at a greater disadvantage than women from other European countries [18]. Some cross-sectional and longitudinal studies confirmed the negative effects of the COVID-19 pandemic on women’s mental health. In a recent study on the general population in Spain, Ausin et al. [12] found significant gender-related differences in the psychological impact of COVID-19, evidencing that women displayed higher levels of depression, anxiety and PTSD, more feelings of loneliness, and less spiritual well-being when compared to men. Similarly, del Río-Casanova et al. [24] found that compared to men, women showed worse psychological adjustment, characterized by a wide range of symptomatology such as anxiety, depression, stress, and posttraumatic symptoms (ranging from mild to moderate). Moreover, a longitudinal study on the trajectories of anxiety and depression over the 20 weeks following the lockdown in England [13] found that although most individuals experienced improvements in mental health when lockdown easing measures were introduced, some vulnerable groups such as women, younger adults, and individuals with lower educational levels showed higher levels of anxiety and depression at the start of lockdown, which decreased but persisted after the earlier phases of confinement.

Although several variables have demonstrated a negative impact on mental health in the general population, little is known about which factors had a greater impact in predicting women’s psychological distress during the pandemic. Previous studies [24,25,26] have examined gender differences in psychological distress after the COVID-19 pandemic, and reported several important factors that were associated with a higher level of mental health problems. In a study conducted in China, Yan et al. [25] found that among female participants, risk factors of distress included poorer health, worse local pandemic status, greater desire for knowledge about COVID-19, the problem of diseases during the pandemic, and an inability to work/study. Conversely, calmness of mood compared with the pre-pandemic period and frequent contact with colleagues were found to act as protective factors against psychological distress. Another recent study [26] found that the caregiving burden, domestic violence, and fear of COVID-19 were independently associated with psychological distress among young women, who presented higher suicide rates during the COVID-19 pandemic.

However, although recent findings shed light on relevant determinants of women’s distress during the COVID-19 pandemic, previous studies are limited by their cross-sectional design, emphasizing the need for further longitudinal studies.

Among the psychological factors associated with mental health problems, the way people cope with stressful and unpredictable events was shown to play a crucial role in reducing or heightening psychological distress during the current pandemic [27,28]. For instance, as demonstrated by Parra-Rizo and Sanchìs-Soler (2021) [29], regular physical activity maintains and enhances physical and psychological functioning, facilitating functional independence as well as the absence of diseases in older women. Relevant previous studies have evidenced how the association between emotional regulation strategies and intolerance of incertitude highlighted the negative effects of uncertainty on affect [30,31]. Specifically, a recent study reporting findings from an online survey during initial COVID-19 lockdowns in the United States [32] suggested that the intolerance of uncertainty may impact mental health by reducing the use of adaptive emotion regulation and implementing maladaptive ones. On the other hand, social support and cognitive reappraisal seem to represent protective factors for mental health [7], jointly with perceived coping efficacy, trust in institutional responses to the COVID-19 emergency, perceived house size, and media exposure to the COVID-19 outbreak as citizens’ positive and negative mental health predictors [33].

### The Present Study

Based on previous studies which showed that women had the highest prevalence of mental health problems during the first period of the COVID-19 pandemic [16,17], the aims of the present study were twofold: (a) to evaluate changes in women’s psychological distress between the first (T1) and the second phases (T2) of the lockdown in Italy (April–May 2020), and (b) to evaluate the role of certain relevant demographic, health, and psychological factors in predicting women’s psychological distress. Thus, the following hypotheses were tested:

**Hypothesis 1 (H1).**   *Consistently with previous studies, suggesting that women’s levels of anxiety and depression persisted after the earlier phase of the pandemic [13], we predicted no significant improvement in psychological distress from T1 to T2.*

**Hypothesis 2 (H2).**  *We expected that psychological distress at T2 would be predicted by factors such as demographic and health profile, COVID-19-related factors, intolerance of uncertainty, and emotion regulation strategies.*

## 2. Materials and Methods

### 2.1. Participants and Procedure

Eligible participants were included if they were aged 18 or over, spoke Italian as their first language, and if they were able to access an electronic device (e.g., mobile phone, computer, laptop, or tablet) connected to the Internet. Data were collected during a period of national lockdown, in two different waves, between the 7th and 24th April 2020 (during the first phase of the COVID-19 outbreak) and between the 18th and 31st May 2020 (during the second phase when the restrictive measures were eased). Participants completed a baseline online assessment on the Google Form web platform (https://docs.google.com/forms/u/0/). The study was conducted according to the guidelines of the Declaration of Helsinki, and approved by the Ethics Committee of the University of Palermo.

The study included a total of 893 women (mean age = 36.45; SD = 14.48). At Time 1, data were collected from 2915 women, as part of a larger study on the psychological consequences of the COVID-19 pandemic. Of this initial sample, 490 (16.8%) were not available to complete the survey at T2. Of the remaining 2425 women, 893 (36.8%) participated in the second wave (Time 2). Only women who provided both T1 and T2 data were included in the present study. Participants’ demographic information is reported in Table 1.

### 2.2. Measures

Participants completed an online survey consisting of the following measures:

Demographic and health surveys were used at T1 to collect information on the age, level of education, occupation (0 = unemployed, 1 = employed), presence of pathologies in the previous year (e.g., respiratory, cardiac, chronic; 0 = no, 1 = yes), presence of disabilities (0 = no, 1 = yes), evaluation of individual’s general health conditions (measured on a 5-point Likert-type scale from 1 “excellent” to 5 “deficient”), and previous COVID-19 diagnosis (0 = no, 1 = yes), or COVID-19 diagnosis among parents/family (0 = no, 1 = yes).

The Depression, Anxiety and Stress Scale (DASS-21) [34,35] is a 21-item self-report scale designed to measure the emotional states of depression, anxiety and stress both at T1 and T2. Participants were asked to indicate the presence of any symptoms over the previous week. Each item is scored from 0 “did not apply to me at all” to 3 “applied to me very much or most of the time”. The higher the score, the higher the levels of depression, anxiety, and stress. The DASS-21 showed good to excellent internal consistency in this study (T1 Cronbach’s alpha = 0.886, 0.868, and 0.914, for depression, anxiety, and stress subscales, respectively. T2 Cronbach’s alpha = 0.899, 0.885, and 0.921 for depression, anxiety, and stress subscales, respectively).

The Emotion Regulation Questionnaire (ERQ) [36,37] was used at T1 to measure individuals’ tendency to regulate their emotions through two strategies: (1) Cognitive Reappraisal (i.e., attempts to reinterpret an emotion-eliciting situation in a way that alters its meaning and changes its emotional impact; six items, e.g., “When I want to feel less negative emotions, I change the way I’m thinking about the situation”), and (2) Expressive Suppression (i.e., attempts to reduce or inhibit ingoing emotion-expressive behavior; four items, e.g., “I control my emotions by not expressing them”). Respondents answered each item on a 7-point Likert-type scale, ranging from 1 “strongly disagree” to 7 “strongly agree”. The higher the score, the greater the use of that regulation strategy. Cronbach’s alpha was 0.886 for the Cognitive Reappraisal subscale, and 0.691 (mean inter-item correlation = 0.357) for the Expressive Suppression subscale.

The Intolerance of Uncertainty Scale-12 (IUS) [38,39] is a 12-item self-report questionnaire (e.g., “When things happen suddenly, I get very upset”) used to measure the dispositional inability of an individual to tolerate uncertainty at T1. The IUS-12 covers two domains (i.e., Prospective and Inhibitory) and also provides a total score to evaluate the general intolerance of uncertainty. Respondents are asked to rate the extent to which each statement applies to themselves on a 5-point Likert scale, ranging from 1 ”not at all characteristic of me” to 5 “entirely characteristic of me”. For the present study, only the total score was used, and it showed good internal consistency (Cronbach’s alpha = 0.887).

### 2.3. Statistical Analyses

As a preliminary step in the data analysis, the normality of continuous variables was checked, and all variables had a normal distribution (Sk: −0.533; 1.566; Ku: −1.001; 2.023). The internal consistency of the scales (Cronbach’s α) was computed, and the mean inter-item correlation was examined for the ERQ Expressive Suppression subscale. Mean inter-item correlations between 0.15 and 0.50 indicated adequate internal consistency [40]. Descriptive statistics for continuous (i.e., means and standard deviations) and qualitative variables (i.e., frequencies and percentages) were computed.

To test the first hypothesis of the study, differences in depression, anxiety, and stress (DASS-21) levels between T1 and T2 were tested using a paired samples t-test. Cohen’s d effect sizes were also reported.

To test the second hypothesis of the study, data at T1 were summarized by principal component analysis (PCA) with Promax oblique rotation. The applicability of the data for the analysis was verified. The case-to-variable ratio was 81.2 (which exceeds the recommended minimum of 10) [41]. The Kaiser–Meyer–Olkin sampling adequacy measure was 0.587 (Hair et al. [42] suggest accepting a value >0.5), and Bartlett’s test of sphericity was significant (*p* < 0.001). The number of factors to retain was determined using visual inspection of the scree plot and an examination of eigenvalues (i.e., factors with eigenvalues greater than 1 were retained). Moreover, multiple regression was computed with factors at T1 as independent variables, and DASS-21 subscales at T2 as dependent variables. Statistical analyses were performed using the Statistical Package for Social Sciences (SPSS), version 22 (IBM Corp., Armonk, NY, USA).

## 3. Results

### 3.1. Changes in Psychological Distress between T1 and T2

Table 2 shows descriptive statistics and changes in psychological distress between T1 and T2. At T2 (after the lockdown phase), women reported significantly lower levels of depression, but with a small effect size. No differences between T1 and T2 were found in anxiety and stress levels, nor in the total DASS-21 score.

### 3.2. Factors Related to Women’s Psychological Distress

As a preliminary step, PCA was used to summarize demographic and health factors, emotion regulation strategies, and intolerance of uncertainty. Both eigenvalues and the visual inspection of the scree plot resulted in the retention of four factors, accounting for 52% of the total variance. Table 3 shows the rotated component matrix with the communalities. The first factor, which explained 16% of the variance, contained three variables and was labeled “Social Stability Status” because it contained demographic data (i.e., age, occupation, and level of education) which are generally related to greater economic security and a more stable social role; the second factor (which explained 14% of the variance) contained three variables and was labeled “Medical Impairment” because it refers to the presence of health diseases unrelated to the COVID-19 pandemic; the third factor (which explains 11% of the variance) contained three variables and was labeled “Maladaptive emotional coping” because it contained data regarding an inability to tolerate uncertainty and maladaptive strategies to regulate emotions; finally, the fourth factor (which explains 10% of the variance) contained two variables and was labeled “Personal contact with COVID-19” because it referred to COVID-19 diagnosis among participants and/or their relatives.

Subsequently, the contribution of these factors was estimated in order to explain stress, depression, and anxiety levels at T2 (Table 4). A higher social stability status at T1 predicted lower stress, depression, and anxiety levels at T2, whereas higher levels of maladaptive emotional coping at T1 predicted higher stress, depression, and anxiety levels at T2. No significant relationships were found between medical impairment and personal contact with COVID-19 factors and dependent variables. There was no multicollinearity among explanatory variables (maximum variance inflation factor: VIF = 1.033).

## 4. Discussion

The present study surveyed a sample of adult women in two different pandemic waves, between the 7 and 24 April 2020 (T1; during the first phase of the COVID-19 lockdown) and between the 18th and 31st May 2020 (T2; during the second phase of the pandemic, when the restrictive measures were eased) to examine psychosocial variables associated with women’s heightened psychological distress. More specifically, we examined changes in depression, anxiety, and stress levels between T1 and T2 and whether demographic, health, and psychological factors predicted women’s psychological distress. To the best of our knowledge, this is the first longitudinal study that has examined risk and protective factors of psychological distress in a large-scale national sample of adult women.

In line with our first hypothesis, no significant improvement in the anxiety and stress levels was found from T1 to T2. This finding is in line with certain studies that evidenced a stable pattern of results for psychological distress during the COVID-19 confinement [13], affecting specific at-risk groups such as women [43]. Moreover, Di Blasi et al. [27], in a study with an Italian sample, showed that depression, stress, and anxiety levels represented a “contiguous pattern”, which remained stable during the earlier phases of the COVID-19 pandemic.

Additionally, previous studies reported that negative impacts of the pandemic on wellbeing have been particularly hard on women, young people, those in lower-income groups, or those who experienced a loss of income [5,44,45].

In the current study, women reported significantly lower levels of depression at T2 (after the lockdown phase), but the effect size was negligible. The slight improvement in our sample seems to corroborate previous studies. For example, Fancourt et al. [13] reported a significant decrease in levels of depression and anxiety over the first 20 weeks following the introduction of a lockdown in England for most individuals, but not for women. Women seem to remain the less recovered and most vulnerable group.

Our second hypothesis was partially supported. More specifically, our results showed that lower social stability status and higher maladaptive emotional coping at T1 predicted higher stress, depression, and anxiety levels at T2. However, no significant relationships were found between “Medical Impairments” and “Personal contact with COVID-19” factors, and dependent variables.

The role of the “Social Stability Status” (i.e., higher age, stable occupation and higher level of education), is consistent with previous studies [5,44,45] and evidenced how the compresence of being young, without a stable paid occupation, and with a low level of education may represent risk factors for psychological distress in women. However, on the other hand, this result indirectly shed light on how the onset of the COVID-19 pandemic has exacerbated existing gender inequalities—namely, in the sphere of economic stability and the gendered divisions of labor—and indicates the need to broaden the focus of research on gender inequalities [46].

Contrary to expectations, no significant relationships were found between “Medical Impairments”, “Personal contact with COVID-19” factors, and perceived psychological distress. This result may support the hypothesis that during the early phase of the pandemic, diseases unrelated and/or related to the COVID-19 pandemic might not have significantly affected psychological distress, whereas the socio-demographic and psychological variables have played a central role in predicting a protective role against unexpected circumstances such as COVID-19.

Additionally, from our analyses, higher “Maladaptive Emotional Coping” (i.e., inability to tolerate uncertainty and maladaptive strategies to regulate emotions) is a predictor of higher psychological distress. One of the mechanisms through which suppression and reappraisal have opposite effects on general wellbeing is their association with positive and negative emotions [47,48]. For example, the use of suppression has been associated with decreases in positive impacts and increases in negative impacts [47]. The use of emotion suppression reflects the association between suppression and psychopathology, e.g., symptoms of anxiety and depression [49]. The findings that the inability to tolerate uncertainty and suppression were associated with higher psychological distress is in line with the prediction that unexpected circumstances, such as a pandemic, demand the fostering of skills to regulate negative emotions in tackling distress [50]. Moreover, this result is consistent with those of a recent COVID-19 study [32] which found that an intolerance of uncertainty affects mental health by reducing the use of adaptive emotion regulation.

Furthermore, the present results are significant, considering that a recent study demonstrated that reappraisal interventions could help to increase psychological resilience and alleviate adverse impacts on women, caused by lockdown and self-isolation [51].

### Strengths and Limitations

The strengths of this study comprise the use of a longitudinal design and the assessment of modifiable psychological factors, such as emotion regulation. A further strength is the recruitment of individuals from a country (i.e., Italy) which has been most dramatically affected by COVID-19. Nonetheless, findings are limited by potential residual, confounding factors and by the requirement to access a mobile device and possess mobile technology knowledge in order to participate. These criteria might represent an obstacle for participation to those from a less advantageous background and limit the inclusivity of the study. Secondly, the study used a non-random population sample; thus, future studies on more representative samples are needed. Thirdly, the results of this study may have been influenced by Italian social–political–cultural patterns that might limit generalizability to other countries. More specifically, the psychological distress of Italian women during the pandemic may have been exacerbated by social inequalities concerning gender. Previous studies have showed that, in Italy, the percentage of those working in professions with a high risk of infection is higher in women than in men [52]; Italian women have also largely been excluded from participating in decision-making regarding the management of the pandemic and post-pandemic recovery [18]; and since the pandemic began, Italian women have reported increased housework and childcare responsibilities (also due to the inability to access external help as a consequence of the lockdown), and have experienced greater job loss, underemployment and precarious positions in the labor market [18]. Additionally, the high dropout rate is a limitation of this study. This could have been due to the length of the questionnaire and the fact that participants did not receive any compensation for participating in the research. Lastly, the time gap between T1 (during the lockdown phase) and T2 (when the lockdown restrictions were eased) might have been too short to detect a significant change in the individuals’ level of psychological distress. Thus, further studies with data collected across multiple time points are needed to confirm our findings.

## 5. Conclusions

Overall, this study showed that psychological distress in a sample of women remained stable over time, with a trivial decrease in depression from T1 to T2. We also found that a higher social stability status and lower maladaptive emotional coping at T1 predicted lower psychological distress at T2. In this context, the social stability status seems to be associated with a protective role against psychological distress, whereas maladaptive emotional coping can be considered a risk factor for women. Women seem to be at greater risk when coping with negative consequences linked to the COVID-19 outbreak; maladaptive emotion regulation strategies (such as suppression in combination with higher inability to tolerate uncertainty and reduced use of cognitive reappraisal) fostered the vulnerability of the women’s group at the time of the first two waves of COVID-19. In summary, the empirical findings of this study provide a new understanding of women’s psychological suffering during the novel pandemic, showing how the socio-demographic and psychological variables play a protective role in the face of unexpected circumstances such as COVID-19.

The results of the study have some clinical and social implications. By dealing with the mechanisms behind self-emotional regulation and tolerance of uncertainty, we might help to cope more promptly with all negative consequences due to a worldwide emergency or economic dissatisfaction. Emotion regulation training might strengthen people’s resilience towards the pandemic’s adverse effects on psychological wellbeing. Specifically, to prevent the chronic manifestations of mental problems, it is necessary to focus psychological preventive and therapeutic interventions on expressing emotions aimed at promoting the use of cognitive reinterpretation of the emotional impact linked to distressing situations. Moreover, policymakers should provide appropriate psychological and social support services to improve women’s emotional well-being, aimed at organizing and increasing resources to support individuals and families both during and after any lockdown measure [53]. Finally, further research is needed in order to focus on the gap in gender differences with regard to the impact of outbreaks across several domains. For example, Etheridge and Spantig (2020) [54] found that women’s well-being during the pandemic was strongly associated with specific dimensions such as family and caring responsibilities, financial and work situations, and social engagements. Interestingly, this study also found that the gender gap in well-being can be explained by gender differences in social factors. Women reported having more close friends before the pandemic than men, and increased loneliness and well-being declined after the pandemic’s onset. This result suggests that lockdown might have impacted women’s mental well-being through a strongly adverse and unequal effect of the direct loss of social interaction.

Overall, results from the present study suggest that policymakers should limit the duration of lockdown and social distancing measures, and they should pay particular attention to at-risk groups, mitigating the impact of lockdown on people’s social and professional lives as an effective strategy for coping with longer periods.

## Figures and Tables

**Table 1:** Demographics and health-related data.

	Sample *n* = 893
Age, M (SD)	36.45 (14.48)
Educational level, *n* (%)	
13 years of schooling	303 (34.0)
Degree/post-graduate	590 (66.0)
Employment status, *n* (%)	
Unemployed	401 (44.9)
Employed (part-time/full-time)	492 (55.1)
Pathologies in the previous year, *n* (%) yes	66 (7.4)
Diagnosed with a disability, *n* (%) yes	17 (1.9)
Own diagnosis of COVID-19, *n* (%) yes	
COVID-19 among relatives, *n* (%) yes	213 (23.9)

**Table 2:** Descriptive statistics and changes in psychological distress between T1 and T2.

	T1 ^2^		T2 ^3^		T	*p*	Cohen’s d ES ^4^
	M	SD	M	SD			
DASS ^1^—Depression	8.97	7.01	8.59	7.25	1.975	0.049	0.066
DASS ^1^—Anxiety	3.90	4.53	3.75	4.62	1.289	0.198	0.043
DASS ^1^—Stress	9.52	5.62	9.42	5.82	0.639	0.523	0.021
DASS ^1^—Total Score	20.40	13.80	19.98	14.39	1.170	0.243	0.039

^1^ DASS, Depression, Anxiety and Stress Scale; ^2^ T1, First phase of the COVID-19 outbreak (period of national lockdown, between 7 and 24 April 2020); ^3^ T2, second phase (when the restrictive measures, between 18 and 31 May 2020); ^4^ ES, effect size.

**Table 3:** Factor loadings and communalities for the rotated matrix.

	Factors				Communalities
Variables and Factor Name	1	2	3	4	
Factor 1: Social Stability Status					
Occupation	0.809				0.666
Level of education	0.651				0.480
Age	0.659				0.512
Factor 2: Medical Impairment					
General health condition		0.734			0.567
Pathologies		0.704			0.497
Disability		0.632			0.442
Factor 3: Maladaptive Emotional Coping					
Intolerance of uncertainty			0.784		0.619
Cognitive Reappraisal			−0.551		0.381
Expressive Suppression			0.678		0.510
Factor 4: Personal contact with COVID-19					
Own COVID-19 diagnosis				0.679	0.482
COVID-19 diagnosis among relatives				0.715	0.524

**Table 4:** Regression analyses.

	Adjusted R^2^	F	β	*p*
DV: DASS ^1^-stress	0.153	41.198 ***		
Factor 1: Social Stability Status			−0.155	0.000
Factor 2: Medical impairment			0.006	0.855
Factor 3: Maladaptive Emotional Coping			0.341	0.000
Factor 4: Personal contact with COVID-19			−0.048	0.123
DV: DASS ^1^-depression	0.223	64.940 ***		
Factor 1: Social Stability Status			−0.072	0.016
Factor 2: Medical impairments			0.003	0.933
Factor 3: Maladaptive Emotional Coping			0.460	0.000
Factor 4: Personal contact with COVID-19			−0.039	0.197
DV: DASS ^1^-anxiety	0.146	39.080 ***		
Factor 1: Social Stability Status			−0.126	0.000
Factor 2: Medical impairments			0.004	0.887
Factor 3: Maladaptive Emotional Coping			0.349	0.000
Factor 4: Personal contact with COVID-19			−0.010	0.750

^1^ DASS, Depression, Anxiety and Stress Scale; *** *p* < 0.001.

## Data Availability

Data are available from the corresponding authors upon reasonable request.
